# Evaluation of diagnostic efficacy of NRP‐1/CD304 in hematological diseases

**DOI:** 10.1002/cam4.5838

**Published:** 2023-03-25

**Authors:** Yi‐jun Liu, Xiao‐hui Li, Yi‐ling Song, Yi‐chen Zhou, Rong‐zeng Cai, Pei‐dong Chi

**Affiliations:** ^1^ Sun Yat‐sen University Cancer Center Guangzhou Guangzhou 510060 People's Republic of China; ^2^ State Key Laboratory of Oncology in South China Guangzhou Guangzhou 510060 People's Republic of China; ^3^ Collaborative Innovation Center for Cancer Medicine Guangzhou Guangzhou 510060 People's Republic of China; ^4^ Department of Clinical Laboratory Sun Yat‐Sen University Cancer Center Guangzhou Guangdong 510060 People's Republic of China

**Keywords:** AML, B‐ALL, BPDCN, CD304, neuropilin‐1

## Abstract

**Background:**

Previous studies had explored the diagnostic or prognostic value of NRP‐1/CD304 in blastic plasmacytoid dendritic cell neoplasm (BPDCN), acute myeloid leukemia (AML), and B‐cell acute lymphoblastic leukemia (B‐ALL), whereas the expression and application value of NRP‐1/CD304 in other common hematological diseases have not been reported.

**Methods:**

Bone marrow samples from 297 newly diagnosed patients with various hematological diseases were collected to detect the expression of NRP‐1/CD304 by flow cytometry (FCM). The diagnostic efficacy of NRP‐1/ CD304‐positive diseases was analyzed by receiver operating characteristic (ROC) curve, and the area under the ROC curve (AUC) was compared.

**Results:**

In the research cohort, the total positive rate of NRP‐1/CD304 was 14.81% (44/297), mainly distributed in BPDCN (100%, 6/6), B‐ALL (48.61%, 35/72), and AML (4.48%, 3/67), with statistically significant differences (*p* < 0.01). Other diseases, such as T‐cell acute lymphoblastic leukemia (T‐ALL), B‐cell non‐Hodgkin lymphoma (B‐NHL), T/NK‐cell lymphoma and plasma cell neoplasms, did not express NRP‐1/CD304. The AUC of NRP‐1/CD304 was 0.936 (95% CI 0.898–0.973), 0.723 (95% CI 0.646–0.801), and 0.435 (95% CI 0.435) in BPDCN, B‐ALL and AML, respectively. Besides, CD304 was commonly expressed in B‐ALL with BCR‐ABL1 gene rearrangement (*p* = 0.000), and CD304 expression was positively correlated with CD34 co‐expression (*p* = 0.009) and CD10 co‐expression (*p* = 0.007).

**Conclusions:**

NRP‐1/CD304 is only expressed in BPDCN, B‐ALL and AML, but not in other common hematological diseases. This indicates that NRP‐1/CD304 has no obvious diagnostic and follow‐up study value in hematological diseases other than BPDCN, B‐ALL, and AML.

## Graphical abstract

The study was to evaluate the diagnostic efficacy of NRP‐1/CD304 in hematological diseases. We found that NRP‐1/CD304 was only expressed in BPDCN, B‐ALL and AML, but not in other common hematological diseases. This indicates that NRP‐1/CD304 has no obvious diagnostic and follow‐up study value in hematological diseases other than BPDCN, B‐ALL and AML.

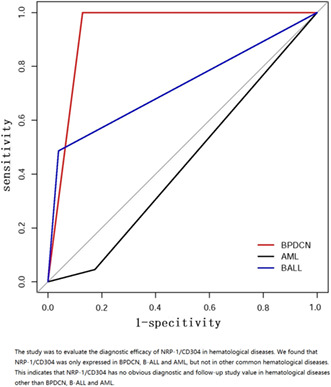

## 1 INTRODUCTION

Neuropilin‐1 (NRP‐1), also known as CD304 (BDCA‐4), is a transmembrane C‐type lectin which was first found on the cell membrane of plasmacytoid dendritic cells (pDCs)^1^; it participates in initiating immune responses and guides dendritic cells into lymphatic ducts.^2^, ^3^ In addition, as a vascular endothelial growth factor receptor (VEGFR), NRP‐1/CD304 plays an important role in angiogenesis and is involved in neuronal guidance during embryogenesis.^4^, ^5^, ^6^, ^7^

Since NRP‐1/CD304 is a useful marker for identifying human pDCs, it has a high sensitivity in the diagnosis of blastic plasmacytoid dendritic cell neoplasm (BPDCN) combined with other markers. Thus, NRP‐1/CD304 is widely used for immunophenotyping detection of BPDCN.^8^, ^9^, ^10^, ^11^, ^12^, ^13^, ^14^, ^15^, ^16^ Furthermore, the vascular effects of NRP‐1/CD304 have led to its investigation in acute leukemia. Some studies demonstrated the expression of NRP‐1/CD304 in acute myeloid leukemia (AML) and B‐cell acute lymphoblastic leukemia (B‐ALL) which applied the methods of reverse transcription‐polymerase chain reaction (RT‐PCR), immunohistochemical staining and flow cytometry (FCM).^17^, ^18^, ^19^, ^20^, ^21^, ^22^ Furthermore, NRP‐1/CD304 has been widely reported to be used in the panel of minimal residual detection (MRD) of B‐ALL. However, there are no literature reports about the expression of NRP‐1/CD304 in other hematological diseases besides BPDCN, AML, and B‐ALL, such as T‐cell acute lymphoblastic leukemia (T‐ALL), B‐cell non‐Hodgkin lymphoma (B‐NHL), T/NK‐cell lymphoma and plasma cell neoplasms. Since these diseases are not uncommon, it makes sense to seek more diagnostic and prognostic indicators. The purpose of this study was to investigate the expression of NRP‐1/CD304 in BPDCN and other common hematological diseases, and to systematically evaluate its diagnostic efficacy, thus to guide the panel design of flow immunophenotyping in diagnosis and MRD of various hematological diseases.

## 2 MATERIALS AND METHODS

### 2.1 Study cohort and clinical data collection

We retrospectively reviewed the flow immunophenotyping results of bone marrow samples from 297 newly diagnosed patients with hematological diseases at Sun Yat‐sen University Cancer Center from October 18, 2013 to April 9, 2021. The study cohort included 183 males and 114 females ranging in age from 0 to 80 years, with a median age of 49 years. There were 6 BPDCN and 291 non‐BPDCN cases in this cohort. The non‐BPDCN cases included 67 AML, 72 B‐ALL, 26 T‐ALL, 67 B‐NHL, 14 T/NK‐cell lymphoma, and 45 plasma cell tumors. Monocytic differentiation was observed in 47 of 67 AML patients. In this study cohort, lymphoma patients underwent bone marrow flow immunophenotyping and histopathological biopsy simultaneously, but not necessarily tissue specimen flow immunophenotyping at the same time. Therefore, bone marrow samples were used to detect the expression of NRP‐1/CD304 by FCM. The classification of B‐NHL and T/NK‐cell lymphoma detected by flow immunophenotyping with bone marrow was based on histopathological biopsy diagnosis of the patient. The subtypes of 67 B‐NHL cases were chronic lymphocytic leukemia/small lymphocytic lymphoma (CLL/SLL) in 38 cases, diffuse large B‐cell lymphoma (DLBCL) and mantle cell lymphoma (MCL) both in seven cases, follicular lymphoma (FL) in six cases, Burkitt lymphoma and hairy cell leukemia (HCL) both in three cases. In addition, there were two cases of mucosal associated lymphoid tissue extranodal marginal zone lymphoma (MALT) and a case of marginal zone lymphoma (MZL). The specific subtypes of 14 cases of T/NK‐cell lymphoma were as follows: six cases of NK/T‐cell lymphoma, three cases of angioimmunoblastic T‐cell lymphoma (AITL), two cases of anaplastic large cell lymphoma (ALCL), one case of Sézary syndrome, T‐cell large granular lymphocytic leukemia (T‐LGL), and peripheral T‐cell lymphoma (PTCL, NOS), respectively. The diagnosis and classification of the diseases referred to the 2016 WHO classification criteria for lymphohematopoietic system tumors^23^, ^24^ and the European Leukemia Immunotyping Group criteria.^25^

All included patients provided informed consent. This study was approved by the ethics committees of Sun Yat‐sen University Cancer Center (SYSUCC, Guangdong, China) (approval number B2022‐362‐01) and was conducted in accordance with the ethical standards of the World Medical Association Declaration of Helsinki. The authenticity of this article has been verified by uploading key raw data to the research data storage public platform (www.researchdata.org.cn), and the approved RDD number is RDDB2021001657.

### 2.2 Diagnostic flow cytometry analysis

Immunophenotyping detection was involved in at least two steps. The first step was to select optimal markers for initial screening based on the patient's history, referring to the Bethesda international consensus recommendations on the immunophenotypic analysis of hematolymphoid neoplasia by FCM.^26^ After having a preliminary idea, the next step of antibody detection was selected according to WHO classification of tumors of hematopoietic and lymphoid tissues 2016.^23^, ^24^ In conclusion, we need to ensure the lineage and maturities of the tumor cells and subtypes to select the antibodies of FCM. After the immunophenotypic results confirmed the diseases to be studied, the expression of target marker NRP‐1/CD304 on abnormal cells was then assessed. Monoclonal antibodies CD303 (clone: AC144) and NRP‐1/CD304 (clone: REA774) were obtained from Miltenyi Biotechnology Company, while other flow antibody reagents were obtained from Becton Dickinson Biosciences or Beckman Coulter. All samples were measured on FACS Canto II FCM (eight colors) and the data files were analyzed using the FACSDiva software (BD Biosciences). The collected bone marrow specimens were anticoagulated with heparin sodium and were detected within 24 h after collection. Samples with severe hemolysis or clots were abandoned. The optimal ratio of cells to antibodies was adjusted by detecting the number of white blood cells in the specimen with a hematology analyzer. Then samples were stained with antibodies for 15 min at room temperature prior to lyse for 15 min in the dark. Lysed cells were washed twice with PBS prior to acquisition.

The descriptions of antibody distribution were negative (<20%), positive (≥80%), or partially expressed (20%–79%) and were relative to an appropriate negative control population. The descriptions of antibody fluorescence intensity were dim, bright, and heterogeneous with the intensity relative to the closest normal hematolymphoid population.^13^, ^26^ According to literature reports, the mean fluorescence intensity (MFI) of plasmacytoid dendritic cells (pDCs) expressing NRP‐1/CD304 in bone marrow was the strongest, and the MFI of mature lymphocytes (CD19‐negative T/NK cells and mature B cells) was the weakest, while the MFI of normal B‐cell precursors was only slightly higher than that of mature lymphocytes.^27^, ^28^, ^29^ Therefore, pDCs and mature B/T/NK were selected as the positive and negative reference cell populations (internal controls) for NRP‐1/CD304, respectively.

### 2.3 Statistical analysis

Statistical analyses were performed using SPSS 24.0 software. The differences in the expression of NRP‐1/CD304 in patients with hematological diseases were determined by the chi‐squared test. The receiver operating characteristic (ROC) curve was used to analyze the diagnostic efficiency of NRP‐1/CD304‐positive diseases, and the area under the ROC curve (AUC) was compared. All tests were two‐sided, and *p ≤*0.05 was considered statistically significant.

## 3 RESULTS

### 3.1 Clinical characteristics of research cohort

A total of 297 patients with newly diagnosed hematological diseases were divided into two groups: BPDCN group (6 cases) and non‐BPDCN group (291 cases). The ratio of male to female in the two groups (BPDCN group was 1:5, non‐BPDCN group 182:109) was statistically significant (*p* = 0.022). The average age of the BPDCN group and the non‐BPDCN group was 34 years (12–70 years) and 50 years (0–80 years), respectively, and there was no significant difference in age (*p* = 0.198) (Table 1).

**TABLE 1:** Age, quantity distribution, and the positive rate of NRP‐1/CD304 in 297 patients with different types of hematological diseases.

Diagnosis	Median (range) age, years	No. of cases	CD304^+^ cases (%)
BPDCN	34 (12–70)	6	6 (100.00)
Non‐BPDCN	50 (0–80)	291	38 (13.06)
AML	48 (5–74)	67	3 (4.48)
B‐ALL	23 (0–62)	72	35 (48.61)
T‐ALL	20 (6–52)	26	0
B‐NHL	55 (4–80)	67	0
T/NK‐cell lymphoma	54 (23–67)	14	0
Plasma cell neoplasms	56 (43–75)	45	0
Total	49 (0–80)	297	44 (14.81)

Abbreviations: AML, acute myeloid leukemia; BPDCN, blastic plasmacytoid dendritic cell neoplasm； B‐ALL, B‐cell acute lymphoblastic leukemia；B‐NHL, B‐cell non‐Hodgkin lymphoma; MDS, myelodysplastic syndrome；T‐ALL, T‐cell acute lymphoblastic leukemia.

### 3.2 NRP‐1/CD304 expression in 297 patients with hematological diseases

The total positive rate of NRP‐1/CD304 in 297 bone marrow specimens was 14.81% (44/297), and the pathological cells of six BPDCN specimens were all expressed NRP‐1/CD304 (6/6, 100%). The total expression rate of NRP‐1/CD304 in non‐BPDCN diseases was 13.06% (38/291) (Table 1). Among non‐BPDCN diseases, the highest positive rate of NRP‐1/CD304 was B‐ALL (48.61%, 35/72), followed by AML (3/67, 4.48%). Two of the three NRP‐1/CD304‐positive AML cases had monocytic differentiation and were diagnosed as acute monoblastic and monocytic leukemia. There was statistical significance in the positive rate of NRP‐1/CD304 in BPDCN, B‐ALL, and AML (*p* < 0.01). In other diseases, such as T‐ALL, B‐NHL, T/NK‐cell lymphoma, and plasma cell tumors, the expression of NRP‐1/CD304 was not detected in the pathological cells.

### 3.3 Clinical characteristics and the expressions of major antigens of 44 cases with NRP‐1/CD304 positive

In the 44 cases of NRP‐1/CD304‐positive patients, the ratio of male to female was 27:17, with an average age of 28 years (0–70 years) (Table S1). In six cases of BPDCN, the positive rate of CD303 was only 50% (3/6), while the expressions of NRP‐1/CD304, CD56, CD4, CD123 and HLA‐DR were all positive, though CD4 was dimly expressed in four BPDCN cases. In 38 cases of non‐BPDCN (including 35 cases of B‐ALL and three cases of AML), CD303 was negatively expressed while NRP‐1/CD304 and HLA‐DR were all positive. The positive rates of CD56, CD4, and CD123 in NRP‐1/ CD304‐positive AML and B‐ALL patients could not be determined in this study since these three markers were not detected in every case.

**FIGURE 1:**
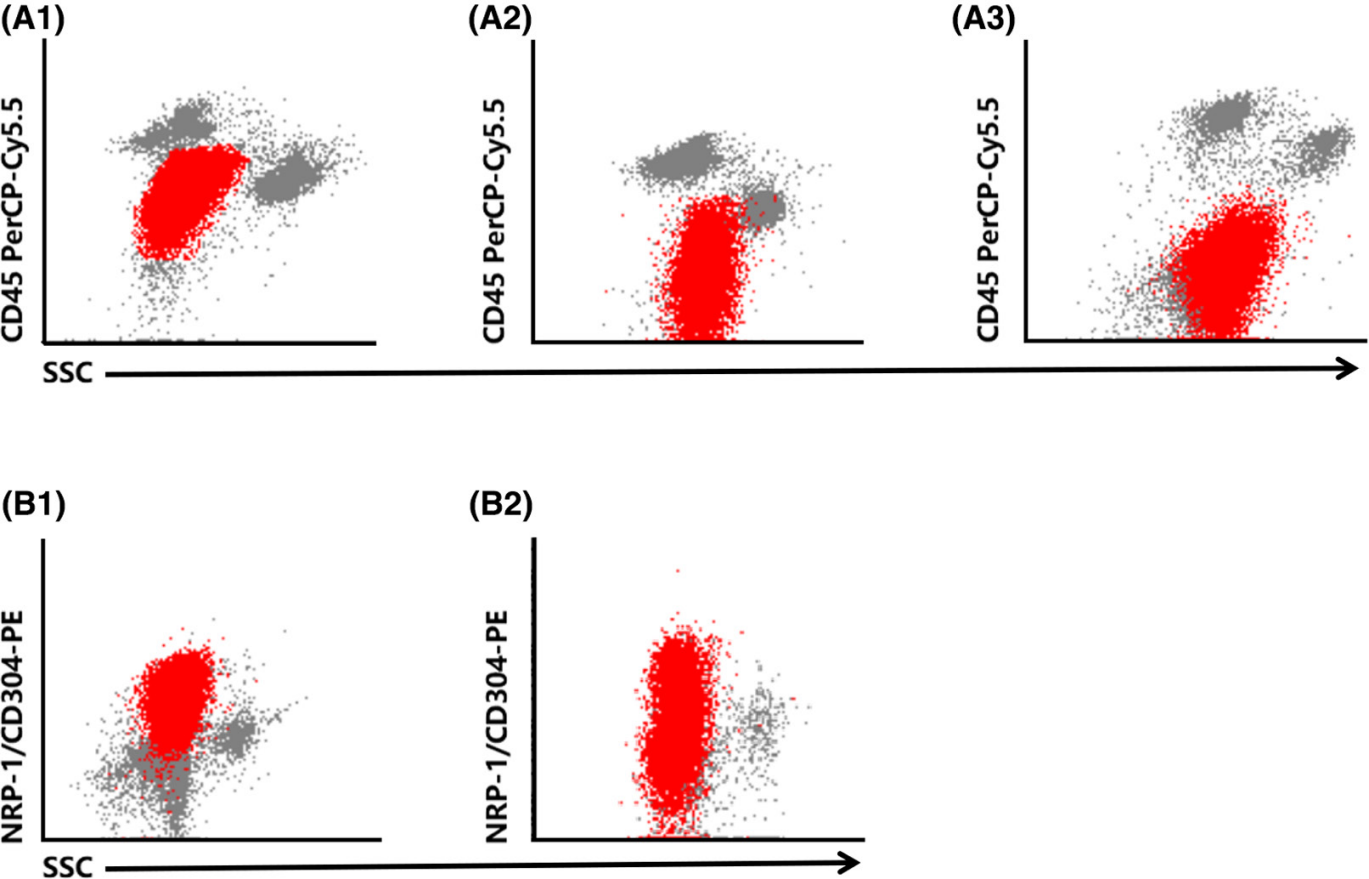
The expression patterns of CD45 and CD304 in B‐ALL patients with NRP‐1/CD304 positive. The populations in red color represent the blastic B‐cell in bone marrow samples which were gated using CD45 and CD19 two‐dimensional dot plot. Row A shows the three expression patterns of CD45. (A1) Dim positive expression. (A2) Dim to negative expression. (A3) Negative expression. Row (B) Two expression patterns of NRP‐1/CD304. (B1) Full expression. (B2) Partial expression.

In addition, we noted that the expression pattern of NRP‐1/CD304 and CD45 was not exactly the same in 35 cases of B‐ALL with NRP‐1/ CD304 positive (Figure 1). Three expression patterns of CD45 are shown in Figure 1A: 18 cases with dim positive expression (51.43%, 18/35) (Figure 1A1), 5 cases with dim to negative expression (14.29%, 5/35) (Figure 1A2) and 12 cases with negative expression (34.29%, 12/35) (Figure 1A3). Two expression patterns of NRP‐1/CD304 are shown in Figure 1B: full expression in 28 cases (80%, 28/35) and partial expression in seven cases (20%, 7/35).

### 3.4 Evaluation of the diagnostic efficacy of NRP‐1/CD304 in BPDCN, B‐ALL, and AML

**FIGURE 2:**
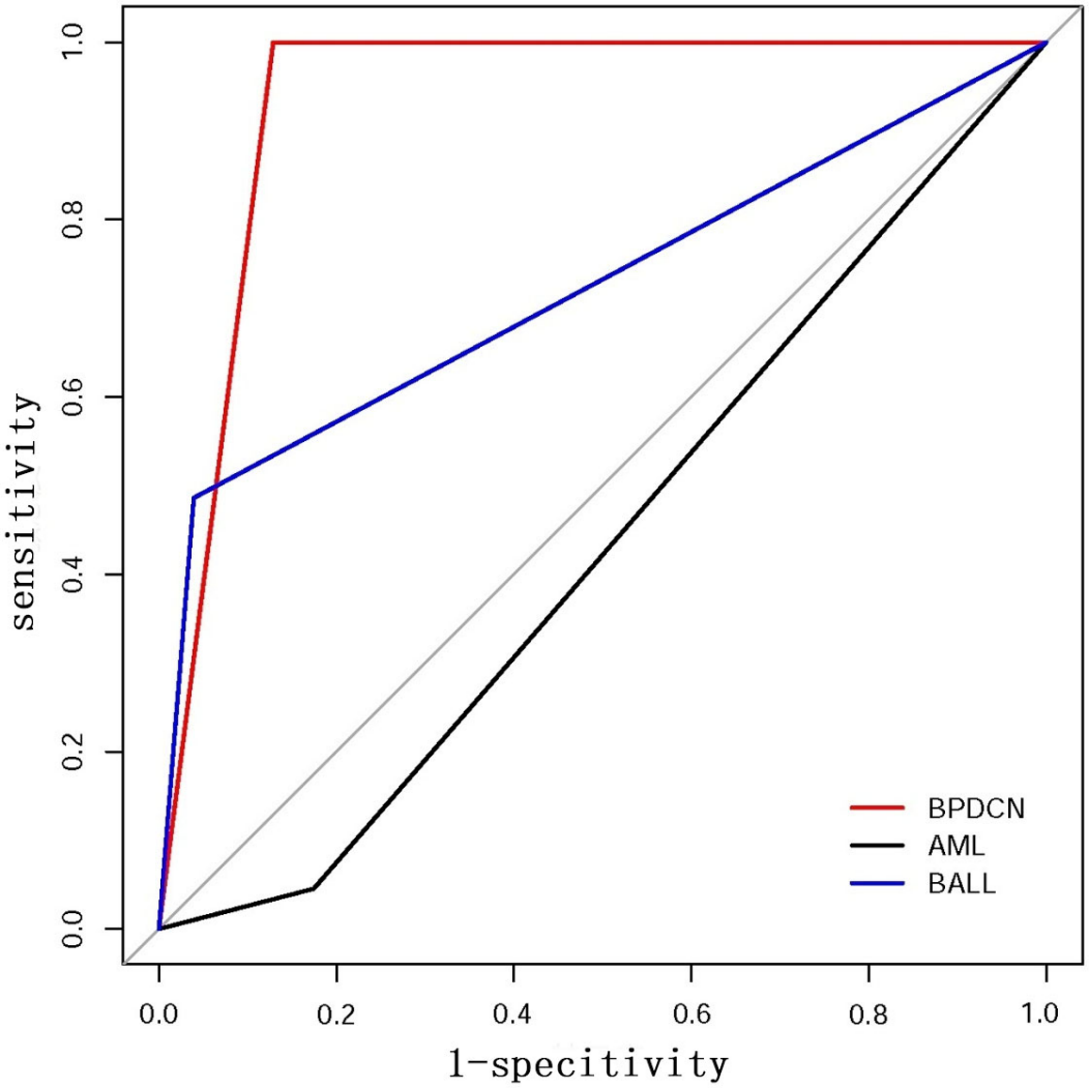
ROC curve analysis of NRP‐1/CD304 for detecting BPDCN, B‐ALL and AML. AML, acute myeloid leukemia; BPDCN, blastic plasmacytoid dendritic cell neoplasm; B‐ALL, B‐cell acute lymphoblastic leukemia. ROC; receiver operating characteristic curve.

Based on the expression of NRP‐1/CD304 in BPDCN, B‐ALL, and AML, we further calculated its diagnostic efficacy in these three diseases. The results showed that the sensitivities of NRP‐1/CD304 for BPDCN, B‐ALL, and AML were 100.00%, 48.61%, and 4.48%, and the specificities were 87.12%, 96.01% and 82.48%, respectively (Table 2). The positive predictive value (PPV) of NRP‐1/CD304 for B‐ALL (79.55%) was much higher than that of BPDCN (13.64%) and AML (6.82%). The negative predictive value (NPV) of NRP‐1/CD304 for BPDCN was 100%, followed by B‐ALL (85.60%) and AML (75.10%). The ROC curve illustrated that the AUC of NRP‐1/CD304 for the diagnosis of BPDCN, B‐ALL, and AML was 0.936 (95% CI 0.898–0.973), 0.723 (95% CI 0.646–0.801), and 0.435 (95% CI 0.362–0.509), respectively (Table 2, Figure 2).

**TABLE 2:** Evaluation of diagnostic efficacy of NRP‐1/CD304 in three hematological diseases with positive expression.

Disease	Sensitivity (%)	Specificity (%)	PPV (%)	NPV (%)	AUC	95% CI	SE	*p* value
BPDCN	100.00	87.12	13.64	100.00	0.936	0.898–0.973	0.019	0.000
B‐ALL	48.61	96.01	79.55	85.60	0.723	0.646–0.801	0.039	0.000
AML	4.48	82.48	6.82	75.10	0.435	0.362–0.509	0.038	0.109

Abbreviations: AUC, area under the curve; AML, acute myeloid leukemia; BPDCN, blastic plasmacytoid dendritic cell neoplasm; B‐ALL, B‐cell acute lymphoblastic leukemia; NPV, negative predictive value; PPV, positive predictive value; SE, standard error; 95% CI, 95% confidence interval.

### 3.5 Correlation analysis between B‐ALL related genes and CD304 expression

B‐ALL related genes ETV6‐RUNX1, BCR‐ABL1, and TEL‐AML1 were detected in 33, 41, and 33 cases of 72 B‐ALL patients, respectively. ETV6‐RUNX1, BCR‐ABL1, and TEL‐AML1 were detected in 0% (0/16), 57.1% (12/21), and 6.3% (1/16) of CD304‐positive B‐ALL cases, respectively. However, the positive rates of these genes were 100% (17/17), 0% (0/20) and 0% (0/17) in CD304‐negative B‐ALL cases, respectively. The results showed that CD304 was commonly expressed in B‐ALL with BCR‐ABL1 gene rearrangement (*p* = 0.000). However, no correlation was found between CD304 expression and other gene rearrangement (Table 3).

**TABLE 3:** Correlative analysis of B‐ALL‐related genetics with CD304 expression.

	ETV6‐RUNX1	BCR‐ABL1	TEL‐AML1
CD304+	0% (0/16)	57.1% (12/21)	6.3% (1/16)
CD304‐	100% (17/17)	0% (0/20)	0% (0/17)
*p* Value	NA	0.000	0.310

Abbreviation: NA, not applicable.

### 3.6 Correlation analysis between B‐ALL related markers and CD304 expression

we investigated the correlation between CD304 expression and B‐ALL related markers, including CD19, CD34, CD10, CD20, CD13, CD33, CD38, TdT, cIgM, CD22, and HLA‐DR. The results showed that CD304 expression was only positively correlated with CD34 co‐expression (*p* = 0.009) and CD10 co‐expression (*p* = 0.007). Details are shown in Table 4.

**TABLE 4:** Correlative analysis of B‐ALL related markers with CD304 expression.

	CD19	CD34	CD10	CD20	CD13	CD33	CD38	TdT	cIgM	CD22	HLA‐DR
CD304 + (*n* = 35)	35 (100.00)	33 (94.30)	35 (100.00)	12 (34.30)	7 (20.00)	12 (34.30)	34 (77.10)	34 (97.10)	4 (11.40)	34 (97.10)	29 (82.90)
CD304‐(*n* = 37)	36 (97.30)	26 (70.30)	30 (81.10)	9 (24.30)	11 (29.70)	9 (24.30)	33 (86.50)	33 (89.20)	10 (27.00)	35 (94.60)	35 (94.60)
*p* value	0.331	0.009	0.007	0.356	0.344	0.356	0.306	0.188	0.097	0.591	0.116

## 4 DISCUSSION

Since NRP‐1/CD304 plays an important role in angiogenesis^3^, ^4^, ^5^; it is a promising target for antiangiogenesis treatment strategies and many scholars have studied its application in acute leukemia.^17^, ^18^, ^19^, ^20^, ^30^ Kreuter et al. firstly evaluated the role of angiogenic factors in AML by detecting the expression of NRP‐1/CD304 in 76 cases of AML by immunohistochemical analysis. They found that NRP‐1/CD304 was overexpressed in all cases compared with normal bone marrow, and there was a significant negative correlation between survival and NRP‐1/CD304 expression. In addition, the authors also found no correlation between the levels of NRP‐1/CD304 expression and AML subtype or karyotype.^17^ Other researchers used FCM to detect the expression of NRP‐1/CD304 in BPDCN, B‐ALL, and they found that NRP‐1/CD304 was positive in varying degrees,^13^, ^22^ which was similar to our study. In our study, the positive rate of CD304 in AML was lower than other literature. Research showed that CD304 expression was very high in M4eo subtype and complex cytogenetics in AML.^22^ However, we did not have M4eo subtype and complex cytogenetics in our study. May be that is why we had different positive rate of CD304 due to the different composition of AML subtypes. It had been verified that NRP‐1/CD304 was a very useful and dependable marker for the MRD assessment of B‐ALL because it was overexpressed in B‐ALL cells compared with normal precursor B cells.^27^, ^28^, ^29^, ^30^ However, there are no comprehensive and detailed reports on the expression of NRP‐1/CD304 in other common hematological diseases besides BPDCN, AML, and B‐ALL, such as T‐ALL, B‐NHL, T/NK‐cell lymphoma, and plasma cell neoplasms. Also, the application value of NRP‐1/CD304 in these diseases is unknown. Therefore, it is necessary to conduct a comprehensive study to evaluate the flow application prospects of NRP‐1/CD304.

According to the guidelines of the literature, pDCs in the specimens were used as the positive expression control of NRP‐1/CD304, and mature lymphocytes were used as the negative expression control. Based on this, we found that the overall positive rate of NRP‐1/CD304 in all specimens was 14.81% (44/297), and the positive rate of NRP‐1/CD304 in descending order was as follows: BPDCN (6/6, 100%), B‐ALL (48.61%, 35/72), AML (3/67, 4.48%), which were similar to those reported in other studies.^22^, ^27^, ^29^, ^31^ Nevertheless, NRP‐1/CD304 was not positive in other hematolymphoid neoplasms (T‐ALL, B‐NHL, T/NK‐cell lymphoma, and plasma cell neoplasms). These results indicated that NRP‐1/CD304 had no obvious value in flow detection and follow‐up study in the above hematological diseases, and it was not necessary to include NRP‐1/CD304 in the panel design of immunophenotyping and MRD detection protocols. In addition, we also found that NRP‐1/CD304 was not positively expressed on normal granulocytes and monocytes in the specimens, and the intensity of expression was comparable to that of normal precursor B cells (results not shown), which was rarely reported in other literature.

The application value of NRP‐1/CD304 in BPDCN, AML, and B‐ALL was worthy of in‐depth study. Further analysis found that NRP‐1/CD304 had higher sensitivity (up to 100%) but lower specificity (87.12%) in the diagnosis of BPDCN. Although NRP‐1/CD304 only accounted for 13.64% of PPV diagnosed by BPDCN, NPV reached 100%. These results suggested that the positive expression of NRP‐1/CD304 alone cannot determine the differentiation of plasmacytoid dendritic cells, and should be combined with other characteristic PDC immunophenotypes (such as CD4^+^, CD56^+^, CD123^high^, CD303^+^, and lack of lineage‐specific antigens) for comprehensive diagnosis.^10^, ^13^, ^32^, ^33^ However, negative expression of NRP‐1/CD304 strongly suggested that the case could not be diagnosed as BPDCN. This conclusion was consistent with other studies^9^, ^10^, ^11^, ^13^ and our actual experience. Additionally, we found that the PPV of NRP‐1/CD304 for B‐ALL (79.55%) was much higher than that of BPDCN (13.64%) and AML (6.82%), and the NPV for B‐ALL reached 85.60%. These results indicated that NRP‐1/CD304 had a promising value in the diagnosis and therapy monitoring of B‐ALL, which is in agreement with other reports.^27^, ^29^, ^31^ We did not assess the utility of NRP‐1/CD304 for the MRD of B‐ALL as it was beyond the scope of this study. In this study, the ROC curve was used to evaluate the diagnostic efficacy of NRP‐1/CD304‐positive diseases, including BPDCN, B‐ALL, and AML. The results showed that the highest diagnostic efficacy was BPDCN (AUC: 0.936), followed by B‐ALL (AUC: 0.723), and NRP‐1/CD304 had no significant diagnostic value for AML (AUC: 0.435). We noted an imbalance in the number of cases between the BPDCN and B‐ALL groups due to the rare incidence of BPDCN. Sample size of BPDCN would lead to the deviation of sensitivity and specificity. The specificity of CD304 in B‐ALL was very high, possibly due to the very small number of cases of BPDCN. The factors affecting the predictive value (PV) are the prevalence, sensitivity, and specificity of the disease. Therefore, the deviation of sensitivity and specificity caused by the small size of the BPDCN group would directly lead to the deviation of PV. In addition, the PPV would show an upward trend, while the NPV would show a downward trend under the condition of unchanged sensitivity and specificity with the increase in prevalence. Therefore, higher incidence of B‐ALL and lower incidence of BPDCN also affected their respective PPVs. Expanding the sample size of BPDCN can make the statistical results more reliable, whereas the influence of the relatively stable prevalence difference between BPDCN and B‐ALL disease itself on PV cannot be eliminated.

We compared the related markers and genes in CD304‐positive and ‐negative arms, and the results showed that CD304 was commonly expressed in B‐ALL with BCR‐ABL1 gene rearrangement. Research has shown that poor prognosis of BCR‐ABL was detected in two‐thirds of pediatric B‐ALL and is likely to be the reason for the already reported poor survival of childhood ALL in Southeast Asia.^34^ CD304 expression was positively correlated with CD34 co‐expression and CD10 co‐expression, in agreement with a previous report.^27^ Meyerson et al.^22^ reported that CD304 expression in B‐ALL was inversely correlated with CD38 with a very weak positive correlation with CD10. Abaza et al. found similar results.^35^ Both results demonstrated limited correlation of CD304 with most of the surface markers typically used in B‐ALL diagnosis.

The male‐to‐female ratio (1:5) and the median age (34 years) (range 12–70) in the BPDCN group observed in our study is inconsistent with literature reports. Many studies had shown that BPDCN was a rare, male‐predominant hematologic malignancy among older patients.^8^, ^9^, ^14^, ^15^, ^36^ The male‐to‐female ratio was approximately 3.3:1 and the median age at diagnosis was 61–67 years without racial or ethnic predilection.^37^ Although a study based on a comprehensive literature database of cases identified a more equal male to female prevalence among patients younger than 40 years,^38^ there was a cohort bias that led to the female predominance and relatively young age of BPDCN cases in our research, and sample sizes should be increased in the future to reduce statistical deviation.

In summary, our study is the first assessment of the positive rate and diagnostic efficacy of NRP‐1/CD304 in various common hematological diseases with large samples. NRP‐1/CD304 is only expressed in BPDCN, B‐ALL, and AML, but not in other common hematological diseases. This indicates that NRP‐1/CD304 has no obvious diagnostic and follow‐up study value in hematological diseases other than BPDCN, B‐ALL, and AML.

## 5 CONCLUSIONS

Our study clarifies the application value of NRP‐1/CD304 in flow immunophenotypic diagnosis and MRD detection of various hematological diseases. NRP‐1/CD304 can be included in the panel design of FCM diagnosis and MRD detection for BPDCN and B‐ALL, but has little significance for AML. Except for BPDCN, AML, and B‐ALL, NRP‐1/CD304 does not need to be included in the flow detection protocol of other hematological diseases.

## AUTHOR CONTRIBUTIONS

**Yi‐jun Liu:** Formal analysis (lead); methodology (lead); writing – original draft (lead). **Xiao‐hui Li:** Data curation (equal); investigation (lead); methodology (lead); visualization (lead). **Yi‐ling Song:** Data curation (equal); formal analysis (equal); investigation (equal); methodology (equal). **Yi‐chen Zhou:** Data curation (equal); formal analysis (equal). **Rong‐zeng Cai:** Resources (lead). **Pei‐dong Chi:** Conceptualization (lead); resources (lead); supervision (lead); writing – review and editing (lead).

## FUNDING INFORMATION

No specific funding was disclosed.

## CONFLICT OF INTEREST STATEMENT

The authors confirm that there are no conflicts of interest.

## ETHICAL APPROVAL

All included patients provided informed consent. This study was approved by the ethics committees of Sun Yat‐sen University Cancer Center (SYSUCC, Guangdong, China; approval number B2022‐362‐01) and was conducted in accordance with the ethical standards of the World Medical Association Declaration of Helsinki.

## Supporting information


Table S1

## Data Availability

The authenticity of this article had been validated by uploading the key raw data onto the Research Data Deposit public platform (www.researchdata.org.cn), with the approval RDD number as RDDB2021001657.
