# Supplementary material for: Evaluation of diagnostic efficacy of NRP‐1/CD304 in hematological diseases
Source: Cancer Med. 2023 Mar 25;12(10):11284–92. doi: 10.1002/cam4.5838 (PMC10242364; doi:10.1002/cam4.5838)
Supplement: Supplementary file 1 — Table S1 [file CAM4-12-11284-s001.docx]

**Table S1** The clinical characteristics and the expressions of major antigens of 44 cases with NRP-1/CD304 positive

| **Sequence no.** | **Gender** | **Age** | **Diagnosis** | **CD303** | **CD304** | **CD56** | **CD4** | **CD123** | **HLA-DR** | **CD45** |
| --- | --- | --- | --- | --- | --- | --- | --- | --- | --- | --- |
| 1 | Female | 21 | BPDCN | － | ＋ | ＋ | dim | ＋ | ＋ | ＋ |
| 2 | Female | 16 | BPDCN | ＋ | ＋ | ＋ | dim | ＋ | ＋ | ＋ |
| 3 | Female | 35 | BPDCN | － | ＋ | ＋ | dim | ＋ | ＋ | ＋ |
| 4 | male | 70 | BPDCN | － | ＋ | ＋ | ＋ | ＋ | ＋ | ＋ |
| 5 | Female | 12 | BPDCN | ＋ | ＋ | ＋ | ＋ | ＋ | ＋ | ＋ |
| 6 | Female | 34 | BPDCN | ＋ | ＋ | ＋ | dim | ＋ | ＋ | ＋ |
| 7 | male | 15 | B-ALL | － | ＋ | － | － | / | ＋ | ＋ |
| 8 | male | 29 | B-ALL | － | ＋ | － | － | / | ＋ | － |
| 9 | male | 52 | B-ALL | － | ＋ | － | － | / | ＋ | － |
| 10 | male | 13 | B-ALL | － | ＋ | － | － | / | ＋ | ＋ |
| 11 | male | 47 | B-ALL | － | ＋ | － | － | / | ＋ | dim to negative |
| 12 | male | 60 | B-ALL | － | ＋ | － | － | / | ＋ | dim to negative |
| 13 | male | 49 | B-ALL | － | ＋ | － | － | － | ＋ | ＋ |
| 14 | Female | 50 | B-ALL | － | part+ | － | － | / | ＋ | dim to negative |
| 15 | Female | 3 | B-ALL | － | ＋ | － | － | / | ＋ | dim to negative |
| 16 | male | 39 | B-ALL | － | part + | － | － | / | ＋ | － |
| 17 | Female | 6 | B-ALL | － | ＋ | － | － | / | ＋ | － |
| 18 | male | 6 | B-ALL | － | ＋ | － | － | / | ＋ | ＋ |
| 19 | Female | 53 | B-ALL | － | ＋ | － | － | / | ＋ | ＋ |
| 20 | male | 28 | B-ALL | － | ＋ | part + | － | － | ＋ | － |
| 21 | male | 9 | B-ALL | － | ＋ | － | － | / | ＋ | － |
| 22 | Female | 3 | B-ALL | － | ＋ | － | － | / | ＋ | － |
| 23 | male | 19 | B-ALL | － | part + | － | － | / | ＋ | ＋ |
| 24 | Female | 61 | B-ALL | － | ＋ | － | － | / | ＋ | ＋ |
| 25 | male | 25 | B-ALL | － | ＋ | － | － | / | ＋ | ＋ |
| 26 | Female | 52 | B-ALL | － | ＋ | / | － | / | ＋ | ＋ |
| 27 | male | 28 | B-ALL | － | ＋ | / | － | / | ＋ | ＋ |
| 28 | male | 6 | B-ALL | － | part + | － | － | / | ＋ | － |
| 29 | Female | 2 | B-ALL | － | ＋ | dim | － | / | ＋ | ＋ |
| 30 | male | 6 | B-ALL | － | ＋ | － | － | / | ＋ | － |
| 31 | male | 3 | B-ALL | － | ＋ | － | － | / | ＋ | dim to negative |
| 32 | Female | 48 | B-ALL | － | part + | / | / | / | ＋ | ＋ |
| 33 | Female | 41 | B-ALL | － | ＋ | / | / | / | ＋ | － |
| 34 | male | 26 | B-ALL | － | ＋ | / | / | / | / | ＋ |
| 35 | Female | 10 | B-ALL | － | ＋ | － | / | / | ＋ | ＋ |
| 36 | male | 23 | B-ALL | － | part + | / | / | / | － | ＋ |
| 37 | male | 13 | B-ALL | － | part + | － | － | / | ＋ | ＋ |
| 38 | male | 52 | B-ALL | － | ＋ | － | － | / | / | － |
| 39 | male | 24 | B-ALL | － | ＋ | part + | / | / | ＋ | － |
| 40 | Female | 37 | B-ALL | － | ＋ | － | － | / | / | ＋ |
| 41 | male | 0 | B-ALL | － | ＋ | － | － | / | ＋ | ＋ |
| 42 | male | 53 | AML | － | part + | ＋ | / | dim | ＋ | ＋ |
| 43 | male | 24 | AML | － | ＋ | － | － | ＋ | ＋ | ＋ |
| 44 | male | 31 | AML | － | ＋ | ＋ | / | / | ＋ | ＋ |

BPDCN: blastic plasmacytoid dendritic cell neoplasm；B-ALL: B-cell acute lymphoblastic leukemia; AML: acute myeloid leukaemia；“/”means not detected
